# Crystal structure of (*E*)-1-(2-nitro­benzyl­idene)-2,2-di­phenyl­hydrazine

**DOI:** 10.1107/S1600536814016109

**Published:** 2014-08-01

**Authors:** Marcos Flores-Alamo, Ruth Meléndrez-Luévano, José A. Ortiz Márquez, Estibaliz Sansinenea Royano, Blanca M. Cabrera-Vivas

**Affiliations:** a Facultad de Química, Universidad Nacional Autónoma de México, 04510, México DF, Mexico; b Facultad de Ciencias Químicas, Benemérita Universidad Autónoma de Puebla 72570, Puebla, Pue., Mexico

**Keywords:** crystal structure, hydrazine, hydrogen bonding

## Abstract

The title compound, C_19_H_15_N_3_O_2_, shows an *E* conformation of the imine bond. The dihedral angle between the planes of the phenyl rings in the di­phenyl­hydrazine groups is 88.52 (4)°. The 2-nitro­benzene ring shows a torsion angle of 10.17 (8)° with the C=N—N plane. A short intra­molecular C—H⋯O contact occurs. In the crystal, only van der Waals contacts occur between the mol­ecules.

## Related literature   

For background to hydrazide–hydrazone derivatives and their various biological activities, see: Sztanke *et al.* (2007 ▶); Al-Macrosaur *et al.* (2007 ▶); Roma *et al.* (2000 ▶); Smalley *et al.* (2006 ▶). For a related structure, see: Mendoza *et al.* (2012 ▶).
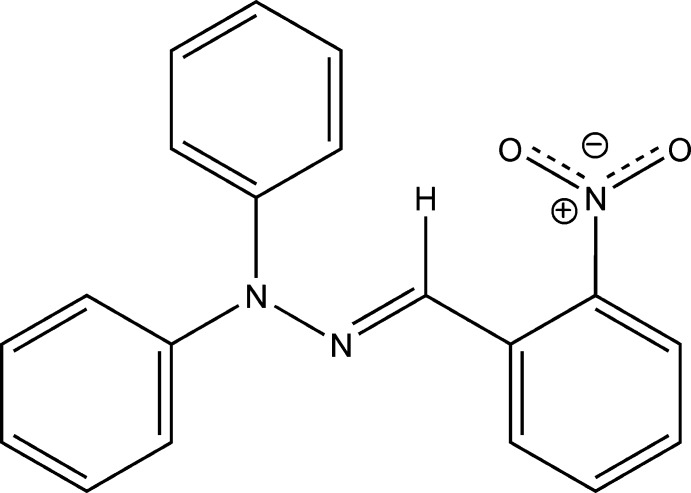


## Experimental   

### Crystal data  

C_19_H_15_N_3_O_2_
*M* *_r_* = 317.34
Monoclinic, 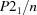

*a* = 11.8536 (5) Å
*b* = 12.4293 (3) Å
*c* = 11.9492 (5) Å
β = 118.584 (5)°
*V* = 1545.92 (12) Å^3^
*Z* = 4
Mo *K*α radiation
μ = 0.09 mm^−1^
*T* = 140 K
0.59 × 0.49 × 0.27 mm

### Data collection  

Agilent Xcalibur Atlas Gemini diffractometer
Absorption correction: analytical (*CrysAlis RED*; Agilent, 2012 ▶) *T*_min_ = 0.961, *T*_max_ = 0.977
12199 measured reflections
3757 independent reflections
3057 reflections with *I* > 2σ(*I*)
*R*_int_ = 0.023

### Refinement  

*R*[*F* ^2^ > 2σ(*F* ^2^)] = 0.042
*wR*(*F* ^2^) = 0.113
*S* = 1.03
3757 reflections
217 parameters
H-atom parameters constrained
Δρ_max_ = 0.19 e Å^−3^
Δρ_min_ = −0.29 e Å^−3^

### 

Data collection: *CrysAlis PRO* (Agilent, 2012 ▶); cell refinement: *CrysAlis RED* (Agilent, 2012 ▶); data reduction: *CrysAlis RED*; program(s) used to solve structure: *SHELXS97* (Sheldrick, 2008 ▶); program(s) used to refine structure: *SHELXL97* (Sheldrick, 2008 ▶); molecular graphics: *Mercury* (Macrae *et al.*, 2006 ▶); software used to prepare material for publication: *WinGX* (Farrugia, 2012 ▶).

## Supplementary Material

Crystal structure: contains datablock(s) I, New_Global. DOI: 10.1107/S1600536814016109/hb7250sup1.cif

Structure factors: contains datablock(s) I. DOI: 10.1107/S1600536814016109/hb7250Isup2.hkl

Supporting information file. DOI: 10.1107/S1600536814016109/hb7250Isup3.cml

**Fig. 1.**
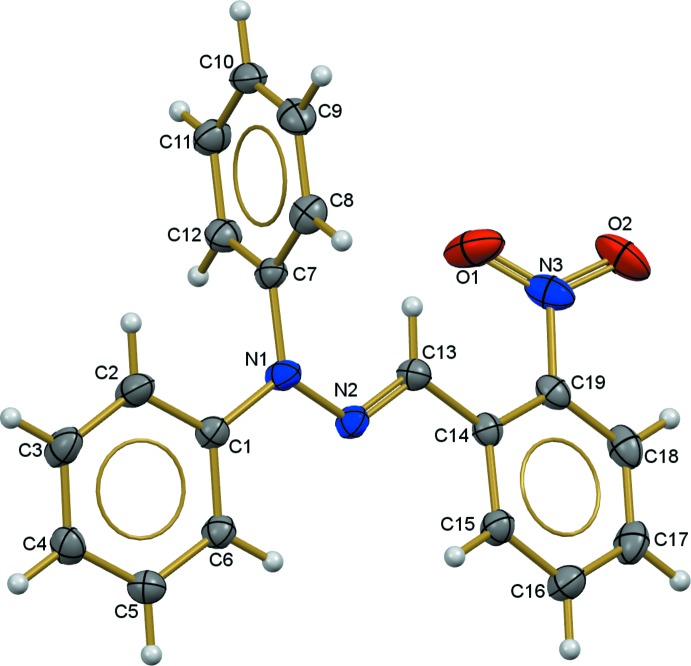
The molecular structure of the title compound. Displacement ellipsoids are drawn at the 50% probability level and H atoms are shown as circles of arbitrary size.

. DOI: 10.1107/S1600536814016109/hb7250fig1.tif
The mol­ecular structure of the title compound. Displacement ellipsoids are drawn at the 50% probability level and H atoms are shown as circles of arbitrary size.

**Fig. 2.**
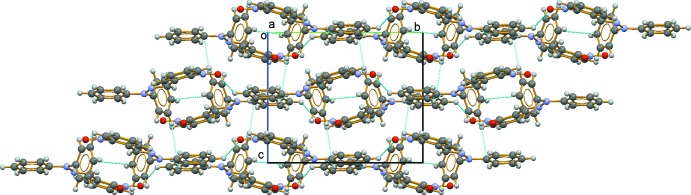
The crystal packing in the title compound.

. DOI: 10.1107/S1600536814016109/hb7250fig2.tif
The crystal packing in the title compound.

CCDC reference: 1013341

Additional supporting information:  crystallographic information; 3D view; checkCIF report

## Figures and Tables

**Table 1:** Hydrogen-bond geometry (Å, °)

*D*—H⋯*A*	*D*—H	H⋯*A*	*D*⋯*A*	*D*—H⋯*A*
C13—H13⋯O1	0.95	2.27	2.7822 (15)	113

## supplementary crystallographic information

### S1. Comment

Hydrazides and hydrazones are present in many of the bioactive heterocyclic compounds that are of great interest because of their diverse biological and clinical applications, creating interest in researchers who have synthesized a variety of hydrazide-hydrazones derivatives and screened them for their various biological activities *viz* anticancer (Sztanke *et al.* 2007), anti-HIV (Al-Macrosaur *et al.* 2007), antimycobacterial, anti-inflammatory (Roma *et al.* 2000), antidiabetic, antimicrobial, as well antimalarial activities (Smalley *et al.* 2006). In the title compound C_19_H_15_N_3_O_2_, the discrete unit consist of one molecule showing an *E* configuration with respect to C=N for diphenylhydrazine group opposite to *o*-nitrophenyl ring (Fig. 1). The dihedral angle for the phenyl rings C1—C6 and C7—C12 is 88.52 (4)° very close to orthogonal form and this value is slightly higher than reported for positional isomer (*E*)-1-(4-nitrobenzylidene)-2,2-diphenylhydrazine (Mendoza *et al.* 2012). The dihedral angle for *ortho*-nitrophenyl ring and C=N—N plane is 10.17 (8) °, which evidences the coplanarity between these groups. The imine N2—C13, 1.2871 (15) Å bond distance is typical C=N bond. In the crystal array one intramolecular interaction C13—H13···O1 (2.27 Å) of type hydrogen bond is observed, and in the crystal packing intermolecular contacts of type van der Waals are observed growing along the *a, b* and *c* axes, resulting in a complex supramolecular array (Fig. 2).

### S2. Experimental

228 mg (1.24 mmol) diphenylhydrazine were dissolved in ethanol and acetic acid (0.5 ml) was slowly added to this solution while stirring, 300 mg (1.24 mmol) of 2-nitrobenzaldehyde was added drop by drop into the above solution strongly stirring and the resulting mixture was kept at room temperature until it became orange transparent solution. After one and a half hours an orange solution precipitated. The reaction was monitored by TLC, aluminium Alugram Sil G/UV254. The mixture was separated with filtration in *vacuo* system and the precipitate was washed three times with cold methanol. Recrystallization was performed three times with ethanol, to obtain orange blocks (yield 91%, mp. 133–135°C). FT·IR (film): (cm^-1^):3026 ν(C—H), 1577 ν(C=N), 1334,ν (NO2). ^1^H NMR (400 MHz, (CD_3_)_2_CO: (d/ p.p.m., *J*/Hz):8.28 (dd,1*H*,C3), 7.91 (dd,1*H*,C5), 7.72 (m,1*H*,C4), 7.60 (s,1*H*,*C*=N),7.52(d, *J* = 1.44, 1H, C6),7.48 (m, 4H,C2') 7.25 (m,6*H*,C4', C2'). ^13^C NMR (400 MHz, (CD_3_)_2_CO): (d/ p.p.m.):143.16 (C2), 132.99 (C1'), 132.97 (C4), 130.41 (C=N), 130.13 (C6), 129.99 (C3'), 128.40 (C1), 127.72 (C3), 125.26 (C4'), 124.49 (C5), 122.41 (C2'). MS—EI: m/*z* 317.12.C_19_H_15_N_3_O_2_.

### S3. Refinement

H atoms bonded to C atoms were placed in geometrical idealized positions and were refined as riding on their parent atoms, with C—H = 0.95 Å with *U*_iso_(H) = 1.2 *U*_eq_(C).

### Crystal data

**Table:**

C_19_H_15_N_3_O_2_	*F*(000) = 664
*M**_r_* = 317.34	*D*_x_ = 1.363 Mg m^−^^3^
Monoclinic, *P*2_1_/*n*	Mo *K*α radiation, λ = 0.71073 Å
Hall symbol: -P 2yn	Cell parameters from 6418 reflections
*a* = 11.8536 (5) Å	θ = 3.7–29.6°
*b* = 12.4293 (3) Å	µ = 0.09 mm^−^^1^
*c* = 11.9492 (5) Å	*T* = 140 K
β = 118.584 (5)°	Block, yellow
*V* = 1545.92 (12) Å^3^	0.59 × 0.49 × 0.27 mm
*Z* = 4	

### Data collection

**Table:**

Agilent Xcalibur Atlas Gemini diffractometer	3757 independent reflections
Graphite monochromator	3057 reflections with *I* > 2σ(*I*)
Detector resolution: 10.4685 pixels mm^-1^	*R*_int_ = 0.023
ω scans	θ_max_ = 29.6°, θ_min_ = 3.7°
Absorption correction: analytical (*CrysAlis RED*; Agilent, 2012)	*h* = −15→15
*T*_min_ = 0.961, *T*_max_ = 0.977	*k* = −17→13
12199 measured reflections	*l* = −12→16

### Refinement

**Table:**

Refinement on *F*^2^	0 restraints
Least-squares matrix: full	H-atom parameters constrained
*R*[*F*^2^ > 2σ(*F*^2^)] = 0.042	*w* = 1/[σ^2^(*F*_o_^2^) + (0.0546*P*)^2^ + 0.3936*P*] where *P* = (*F*_o_^2^ + 2*F*_c_^2^)/3
*wR*(*F*^2^) = 0.113	(Δ/σ)_max_ < 0.001
*S* = 1.03	Δρ_max_ = 0.19 e Å^−^^3^
3757 reflections	Δρ_min_ = −0.29 e Å^−^^3^
217 parameters	

### Special details

**Table:**

Geometry. All e.s.d.'s (except the e.s.d. in the dihedral angle between two l.s. planes) are estimated using the full covariance matrix. The cell e.s.d.'s are taken into account individually in the estimation of e.s.d.'s in distances, angles and torsion angles; correlations between e.s.d.'s in cell parameters are only used when they are defined by crystal symmetry. An approximate (isotropic) treatment of cell e.s.d.'s is used for estimating e.s.d.'s involving l.s. planes.

### Fractional atomic coordinates and isotropic or equivalent isotropic displacement parameters (Å^2^)

**Table:**

	*x*	*y*	*z*	*U*_iso_*/*U*_eq_	
N2	0.11331 (9)	0.25147 (8)	0.92802 (9)	0.0231 (2)	
N1	0.22974 (9)	0.29935 (8)	0.97008 (10)	0.0258 (2)	
C1	0.23071 (11)	0.41296 (9)	0.97290 (10)	0.0222 (2)	
C6	0.11903 (11)	0.47065 (9)	0.94355 (11)	0.0241 (2)	
H6	0.042	0.4335	0.9251	0.029*	
C13	0.10253 (11)	0.14893 (9)	0.91111 (11)	0.0228 (2)	
H13	0.1743	0.1053	0.9263	0.027*	
C5	0.12018 (12)	0.58203 (10)	0.94130 (11)	0.0269 (3)	
H5	0.0434	0.6206	0.9203	0.032*	
C18	−0.18121 (12)	−0.04581 (10)	0.79564 (12)	0.0306 (3)	
H18	−0.1998	−0.1194	0.7728	0.037*	
C14	−0.02595 (11)	0.10233 (9)	0.86714 (10)	0.0212 (2)	
C4	0.23172 (12)	0.63796 (10)	0.96930 (12)	0.0286 (3)	
H4	0.2315	0.7143	0.9655	0.034*	
C9	0.53623 (12)	0.14688 (10)	1.11874 (12)	0.0304 (3)	
H9	0.5981	0.1224	1.2007	0.037*	
C15	−0.12374 (11)	0.16652 (10)	0.86682 (11)	0.0256 (3)	
H15	−0.1055	0.2396	0.8923	0.031*	
C12	0.35602 (11)	0.21828 (10)	0.87944 (11)	0.0267 (3)	
H12	0.2942	0.2428	0.7974	0.032*	
C7	0.34034 (10)	0.23976 (9)	0.98487 (11)	0.0222 (2)	
C2	0.34400 (12)	0.46897 (10)	1.00538 (13)	0.0319 (3)	
H2	0.4216	0.4308	1.0292	0.038*	
C8	0.42978 (11)	0.20381 (10)	1.10458 (11)	0.0275 (3)	
H8	0.4182	0.2181	1.1765	0.033*	
O2	0.00953 (12)	−0.17689 (8)	0.82260 (11)	0.0517 (3)	
N3	0.02964 (11)	−0.08055 (9)	0.81874 (10)	0.0347 (3)	
C19	−0.06012 (11)	−0.00479 (9)	0.82864 (11)	0.0243 (2)	
C16	−0.24502 (12)	0.12733 (11)	0.83091 (12)	0.0307 (3)	
H16	−0.3091	0.1738	0.8299	0.037*	
C10	0.55296 (12)	0.12539 (10)	1.01392 (13)	0.0289 (3)	
H10	0.6262	0.0865	1.0239	0.035*	
C11	0.46250 (12)	0.16084 (10)	0.89447 (12)	0.0302 (3)	
H11	0.4735	0.1457	0.8224	0.036*	
C17	−0.27406 (12)	0.02027 (11)	0.79614 (12)	0.0327 (3)	
H17	−0.3572	−0.0071	0.7729	0.039*	
O1	0.11844 (10)	−0.04545 (9)	0.80403 (12)	0.0537 (3)	
C3	0.34333 (13)	0.58067 (11)	1.00287 (14)	0.0341 (3)	
H3	0.4208	0.6183	1.0246	0.041*	

### Atomic displacement parameters (Å^2^)

**Table:**

	*U*^11^	*U*^22^	*U*^33^	*U*^12^	*U*^13^	*U*^23^
N2	0.0200 (5)	0.0234 (5)	0.0265 (5)	−0.0030 (4)	0.0115 (4)	−0.0025 (4)
N1	0.0186 (5)	0.0219 (5)	0.0368 (6)	−0.0018 (4)	0.0132 (4)	−0.0053 (4)
C1	0.0231 (6)	0.0214 (6)	0.0236 (6)	−0.0019 (4)	0.0125 (4)	−0.0041 (4)
C6	0.0207 (5)	0.0248 (6)	0.0261 (6)	−0.0023 (4)	0.0107 (4)	−0.0017 (4)
C13	0.0219 (6)	0.0217 (6)	0.0240 (6)	0.0016 (4)	0.0102 (4)	−0.0004 (4)
C5	0.0259 (6)	0.0259 (6)	0.0285 (6)	0.0030 (5)	0.0128 (5)	−0.0004 (5)
C18	0.0324 (7)	0.0234 (6)	0.0277 (6)	−0.0069 (5)	0.0077 (5)	−0.0010 (5)
C14	0.0228 (5)	0.0211 (5)	0.0190 (5)	−0.0007 (4)	0.0093 (4)	0.0006 (4)
C4	0.0352 (7)	0.0209 (6)	0.0340 (7)	−0.0026 (5)	0.0200 (6)	−0.0040 (5)
C9	0.0222 (6)	0.0298 (7)	0.0296 (7)	−0.0013 (5)	0.0047 (5)	0.0030 (5)
C15	0.0250 (6)	0.0237 (6)	0.0300 (6)	−0.0029 (5)	0.0147 (5)	−0.0039 (4)
C12	0.0238 (6)	0.0301 (6)	0.0245 (6)	0.0031 (5)	0.0102 (5)	0.0033 (5)
C7	0.0183 (5)	0.0192 (5)	0.0294 (6)	−0.0025 (4)	0.0117 (4)	−0.0035 (4)
C2	0.0250 (6)	0.0274 (6)	0.0483 (8)	−0.0028 (5)	0.0216 (6)	−0.0088 (5)
C8	0.0260 (6)	0.0303 (6)	0.0248 (6)	−0.0040 (5)	0.0110 (5)	−0.0045 (5)
O2	0.0655 (8)	0.0209 (5)	0.0550 (7)	0.0089 (5)	0.0178 (6)	−0.0016 (4)
N3	0.0321 (6)	0.0263 (6)	0.0322 (6)	0.0054 (5)	0.0045 (4)	−0.0066 (4)
C19	0.0259 (6)	0.0213 (5)	0.0210 (5)	0.0012 (5)	0.0074 (4)	0.0007 (4)
C16	0.0251 (6)	0.0342 (7)	0.0353 (7)	−0.0010 (5)	0.0165 (5)	−0.0025 (5)
C10	0.0208 (6)	0.0234 (6)	0.0418 (7)	0.0023 (5)	0.0145 (5)	0.0001 (5)
C11	0.0299 (6)	0.0326 (7)	0.0335 (7)	0.0012 (5)	0.0195 (5)	−0.0016 (5)
C17	0.0251 (6)	0.0369 (7)	0.0330 (7)	−0.0095 (5)	0.0113 (5)	−0.0003 (5)
O1	0.0338 (6)	0.0464 (6)	0.0806 (8)	−0.0013 (5)	0.0271 (6)	−0.0269 (6)
C3	0.0310 (7)	0.0294 (7)	0.0502 (8)	−0.0093 (5)	0.0261 (6)	−0.0103 (6)

### Geometric parameters (Å, º)

**Table:**

N2—C13	1.2871 (15)	C9—H9	0.95
N2—N1	1.3593 (13)	C15—C16	1.3776 (17)
N1—C1	1.4125 (15)	C15—H15	0.95
N1—C7	1.4412 (14)	C12—C7	1.3845 (16)
C1—C2	1.3928 (16)	C12—C11	1.3849 (17)
C1—C6	1.3948 (16)	C12—H12	0.95
C6—C5	1.3849 (17)	C7—C8	1.3851 (16)
C6—H6	0.95	C2—C3	1.3887 (18)
C13—C14	1.4714 (15)	C2—H2	0.95
C13—H13	0.95	C8—H8	0.95
C5—C4	1.3856 (17)	O2—N3	1.2261 (15)
C5—H5	0.95	N3—O1	1.2274 (16)
C18—C17	1.3757 (19)	N3—C19	1.4674 (16)
C18—C19	1.3907 (17)	C16—C17	1.3874 (19)
C18—H18	0.95	C16—H16	0.95
C14—C19	1.4036 (16)	C10—C11	1.3844 (18)
C14—C15	1.4057 (16)	C10—H10	0.95
C4—C3	1.3821 (18)	C11—H11	0.95
C4—H4	0.95	C17—H17	0.95
C9—C10	1.3845 (18)	C3—H3	0.95
C9—C8	1.3846 (17)		
			
C13—N2—N1	119.91 (10)	C7—C12—H12	120.3
N2—N1—C1	116.22 (9)	C11—C12—H12	120.3
N2—N1—C7	121.56 (9)	C12—C7—C8	120.52 (11)
C1—N1—C7	120.95 (9)	C12—C7—N1	119.85 (10)
C2—C1—C6	119.06 (11)	C8—C7—N1	119.63 (10)
C2—C1—N1	120.14 (10)	C3—C2—C1	119.92 (12)
C6—C1—N1	120.80 (10)	C3—C2—H2	120
C5—C6—C1	120.12 (11)	C1—C2—H2	120
C5—C6—H6	119.9	C9—C8—C7	119.57 (11)
C1—C6—H6	119.9	C9—C8—H8	120.2
N2—C13—C14	116.99 (10)	C7—C8—H8	120.2
N2—C13—H13	121.5	O2—N3—O1	123.23 (12)
C14—C13—H13	121.5	O2—N3—C19	117.53 (12)
C6—C5—C4	120.92 (11)	O1—N3—C19	119.22 (11)
C6—C5—H5	119.5	C18—C19—C14	122.52 (11)
C4—C5—H5	119.5	C18—C19—N3	115.57 (11)
C17—C18—C19	119.94 (11)	C14—C19—N3	121.90 (11)
C17—C18—H18	120	C15—C16—C17	120.31 (12)
C19—C18—H18	120	C15—C16—H16	119.8
C19—C14—C15	115.42 (10)	C17—C16—H16	119.8
C19—C14—C13	125.28 (10)	C11—C10—C9	119.72 (11)
C15—C14—C13	119.26 (10)	C11—C10—H10	120.1
C3—C4—C5	118.81 (11)	C9—C10—H10	120.1
C3—C4—H4	120.6	C10—C11—C12	120.40 (11)
C5—C4—H4	120.6	C10—C11—H11	119.8
C10—C9—C8	120.30 (11)	C12—C11—H11	119.8
C10—C9—H9	119.8	C18—C17—C16	119.32 (12)
C8—C9—H9	119.8	C18—C17—H17	120.3
C16—C15—C14	122.45 (11)	C16—C17—H17	120.3
C16—C15—H15	118.8	C4—C3—C2	121.07 (12)
C14—C15—H15	118.8	C4—C3—H3	119.5
C7—C12—C11	119.48 (11)	C2—C3—H3	119.5
			
C13—N2—N1—C1	173.38 (10)	N1—C1—C2—C3	177.32 (11)
C13—N2—N1—C7	6.11 (16)	C10—C9—C8—C7	−0.38 (18)
N2—N1—C1—C2	−175.12 (10)	C12—C7—C8—C9	0.64 (18)
C7—N1—C1—C2	−7.77 (16)	N1—C7—C8—C9	−179.78 (11)
N2—N1—C1—C6	4.95 (15)	C17—C18—C19—C14	2.08 (18)
C7—N1—C1—C6	172.30 (10)	C17—C18—C19—N3	−177.01 (11)
C2—C1—C6—C5	2.89 (17)	C15—C14—C19—C18	−1.66 (16)
N1—C1—C6—C5	−177.17 (11)	C13—C14—C19—C18	175.84 (11)
N1—N2—C13—C14	179.18 (9)	C15—C14—C19—N3	177.37 (10)
C1—C6—C5—C4	−0.71 (17)	C13—C14—C19—N3	−5.13 (17)
N2—C13—C14—C19	171.78 (11)	O2—N3—C19—C18	−22.36 (16)
N2—C13—C14—C15	−10.81 (16)	O1—N3—C19—C18	155.89 (12)
C6—C5—C4—C3	−1.63 (18)	O2—N3—C19—C14	158.54 (11)
C19—C14—C15—C16	−0.18 (17)	O1—N3—C19—C14	−23.21 (17)
C13—C14—C15—C16	−177.84 (11)	C14—C15—C16—C17	1.60 (19)
C11—C12—C7—C8	−0.34 (18)	C8—C9—C10—C11	−0.18 (19)
C11—C12—C7—N1	−179.92 (11)	C9—C10—C11—C12	0.48 (19)
N2—N1—C7—C12	80.11 (14)	C7—C12—C11—C10	−0.23 (19)
C1—N1—C7—C12	−86.56 (14)	C19—C18—C17—C16	−0.59 (19)
N2—N1—C7—C8	−99.47 (13)	C15—C16—C17—C18	−1.19 (19)
C1—N1—C7—C8	93.86 (13)	C5—C4—C3—C2	1.77 (19)
C6—C1—C2—C3	−2.75 (18)	C1—C2—C3—C4	0.4 (2)

### Hydrogen-bond geometry (Å, º)

**Table:**

*D*—H···*A*	*D*—H	H···*A*	*D*···*A*	*D*—H···*A*
C13—H13···O1	0.95	2.27	2.7822 (15)	113
