# Supplementary figures and images for: Crystal structure of (E)-1-(2-nitro­benzyl­idene)-2,2-di­phenyl­hydrazine
Source: Acta Crystallogr Sect E Struct Rep Online. 2014 Aug 1;70(Pt 9):o909–10. doi: 10.1107/S1600536814016109 (PMC4186103; doi:10.1107/S1600536814016109)

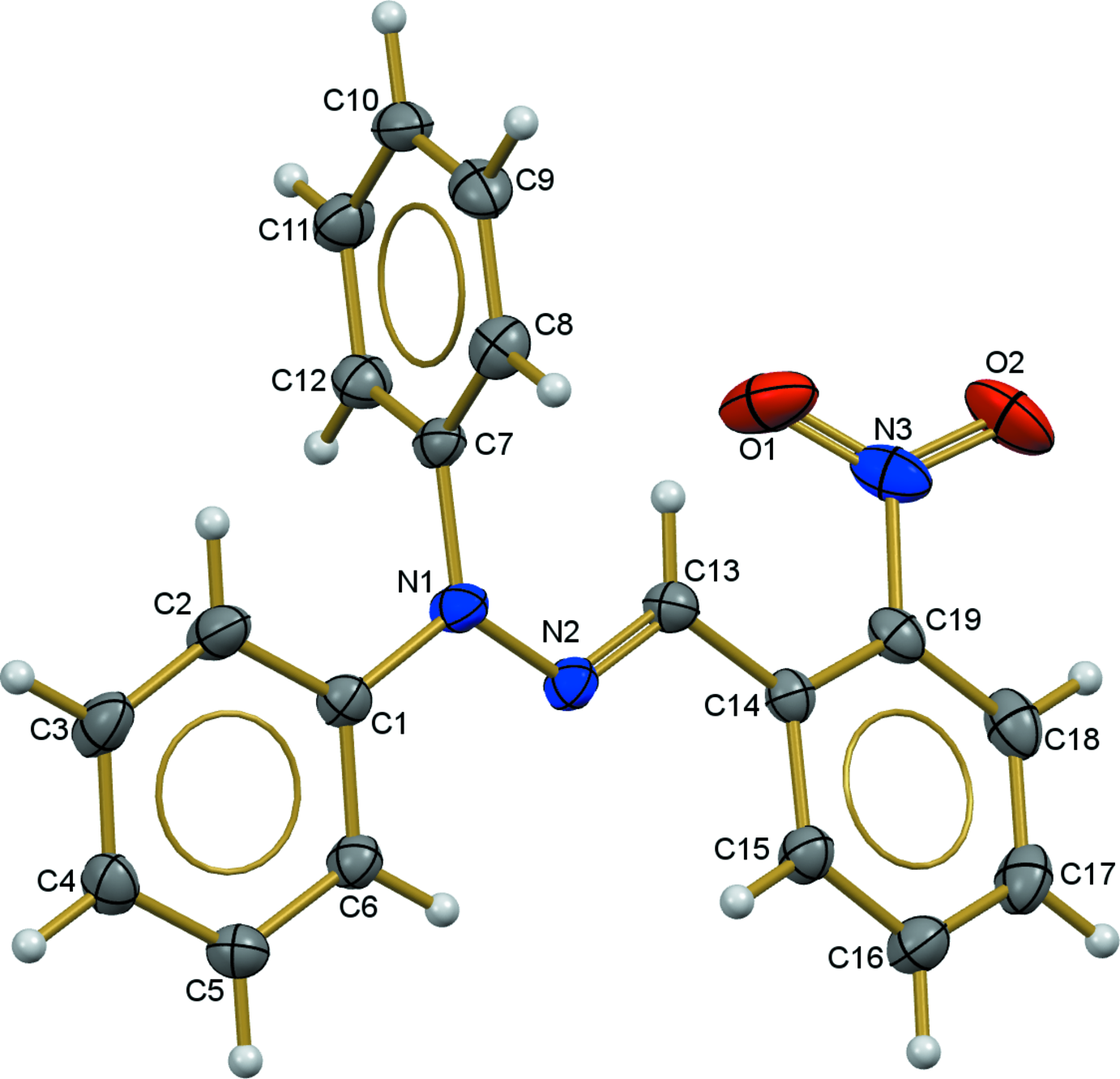

Supplement: Supplementary file 4 [file e-70-0o909-fig1.tif]

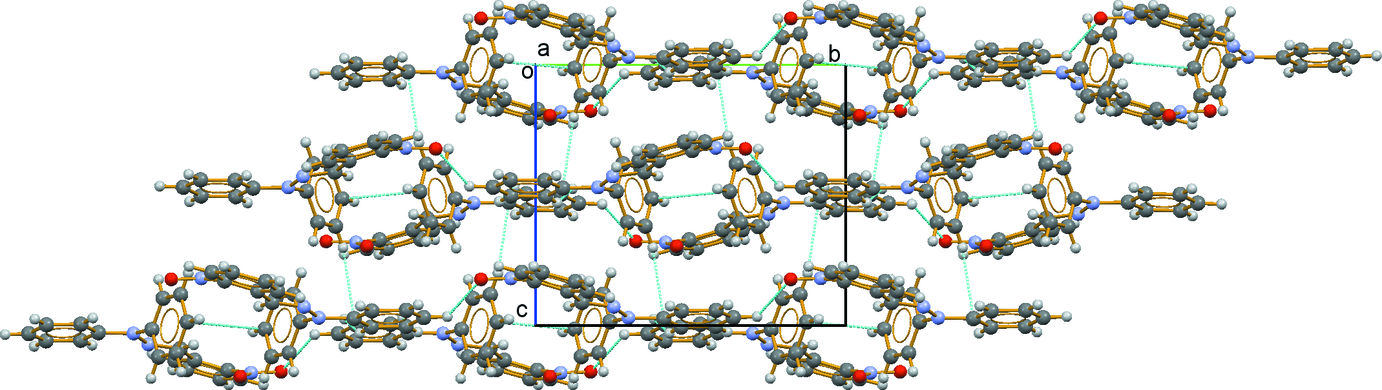

Supplement: Supplementary file 5 [file e-70-0o909-fig2.tif]
